# Integrated delivery of school health interventions through the school platform: Investing for the future

**DOI:** 10.1371/journal.pntd.0006449

**Published:** 2019-01-31

**Authors:** Laura J. Appleby, Gemechu Tadesse, Yonas Wuletawu, Nigussie G. Dejene, Jack E. T. Grimes, Michael D. French, Askale Teklu, Berhanu Moreda, Nebiyu Negussu, Biruck Kebede, Elodie Yard, Iain Gardiner, Lesley J. Drake

**Affiliations:** 1 Partnership for Child Development, Department of Infectious Disease Epidemiology, Imperial College London, London, United Kingdom; 2 Department of Bacterial, Parasitic and Zoonotic Diseases, Ethiopian Public Health Institute, Addis Ababa, Ethiopia; 3 Department of Civil and Environmental Engineering, Imperial College London, London, United Kingdom; 4 Schistosomiasis Control Initiative, Department of Infectious Disease Epidemiology, Imperial College London, London, United Kingdom; 5 United Nations World Food Programme, Addis Ababa, Ethiopia; 6 Federal Ministry of Education, Addis Ababa, Ethiopia; 7 Federal Ministry of Health, Addis Ababa, Ethiopia; Ministère de la Santé Publique et de la Lutte contre les Endémies, NIGER

## Abstract

School health and nutrition (SHN) programmes are recognized as a significant contributor to both health and education sector goals. The school system offers an ideal platform from which to deliver basic health interventions that target the most common health conditions affecting school-age children (SAC) in low-income countries, leading to improved participation and learning outcomes. However, governments require evidence to cost, design, and implement these programmes. In Ethiopia, prevalent health conditions affecting SAC's education participation and learning outcomes include infection with soil-transmitted helminths (STHs), hunger, and malnutrition. In recognition of the multiple issues affecting the health and education of SAC, the government has taken a proactive approach, coordinating an integrated SHN programme designed to be implemented in partnership and monitored and financed through a single, integrated mechanism. The programme, known as the Enhanced School Health Initiative (ESHI), integrates three complimentary health interventions: deworming; school feeding; and provision of a water, sanitation, and hygiene (WASH) package in schools, which in delivery aim to maximize the benefits of each of the individual components. Operational research surrounding the ESHI programme includes both qualitative and quantitative analyses. Here, we present an overview of the ESHI programme and its genesis. We also introduce three additional supporting papers that provide in-depth analyses of key findings, including the baseline situational analysis, the costs, and community perceptions of the programme. The findings from ESHI provide initial evidence to develop an understanding of the related costs and synergies of integrating multiple health interventions onto a single platform. The work has translated into strengthened institutional capacity and improved cross-sectoral coordination. The government is now committed to supporting 25 million school children in Ethiopia through SHN. The ESHI model serves as a reference point for other countries looking to scale up targeted SHN interventions.

## The importance of school health and nutrition to child development

Many health conditions prevalent in school-age children (SAC), such as inadequate food energy consumption, nutritional deficiencies, and infection with parasites such as soil-transmitted helminths (STHs) and schistosomes have important effects on access to education that lead to grade repetition or drop out and can adversely affect student achievement [1–6]. Yet the school-going age group in low- and middle-income countries suffers from a lack of investment from the health sector [7]. These conditions, which are preventable and treatable, have significant impacts on the education sector and are estimated to translate into the equivalent of between 200 to 500 million days of school lost each year [1].

In the recently published Child and Adolescent Health Volume of the third edition of Disease Control Priorities (DCP3), the authors advocate for the collaboration of the health and education sectors in providing operational, human, and financial resources to deliver a package of interventions using existing infrastructure to address the immediate and growing needs of these SAC [7]. The efforts to achieve the Millennium Development Goals (MDGs) have resulted in significant increases in school enrollment. With a global enrollment rate of 91%, more children are attending school today than ever before [8, 9]; as a result, schools provide easy access to this age group for the targeting of health and nutrition interventions.

School-based health and nutrition programmes, collectively known as school health and nutrition (SHN), which target some of the most common health conditions affecting SAC, are among the most ubiquitous forms of health services for SAC in low- and middle-income countries [10, 11]. SHN uses the school as a platform to deliver safe and simple treatments whilst providing health education and protective behaviour change messaging and is advocated for as a key investment of both health and education sectors for achieving increases in school enrollment and attendance while reducing gender gaps [7].

In areas where children are suffering from multiple conditions, integration of multiple interventions onto a single platform may provide a means to optimize programme delivery, maximize efficiencies, and increase the scale and impact of SHN for the health and educational benefit of millions of children in need.

As a first of its kind, an integrated SHN programme was developed in Southern Ethiopia, designed to address the multiple burdens facing SAC in the region. The interventions included deworming to address the burden of helminth infection; hygiene promotion, latrine construction, and provision of a water supply to address the poor access to water and sanitation; and school feeding to address hunger and nutritional deficiencies. We present an overview of the programme here, known as the Enhanced School Health Initiative (ESHI), and introduce three additional papers that present an in-depth analysis of key findings, including the baseline situational analysis, the costs, and the perceptions of the programme.

## The Ethiopian situation

In Ethiopia, 25.3 million SAC are estimated to be living in STH-endemic areas [12], evidence of open defecation is observed in 40% of schools [13], and nearly a quarter of SAC have been reported to be suffering from nutritional-related growth deficiencies or anaemia [13–15]. In recognition of these multiple issues affecting the health and education of SAC in Ethiopia, and of the importance of SHN to health and education, the Ethiopian government has listed SHN as a cross-cutting issue in the new Education Sector Development Plan V and in 2016, launched an SHN strategy based on the Focusing Resources on Effective School Health (FRESH) approach [16–18]. The strategy aims to improve access and educational achievement of school children through health and nutrition interventions and programmes across schools in Ethiopia. The objectives are to promote joint planning, designing, and implementation for sustainable health and nutrition interventions across the education sector and to strengthen coordination, linkage, and partnership of SHN interventions by relevant ministries and other stakeholders [17].

There are numerous ongoing SHN initiatives in Ethiopia, including school feeding [16]; school-based deworming and neglected tropical disease control more generally [12, 19]; and water, sanitation, and hygiene (WASH) interventions [20]. These different components are interrelated: the impact of school feeding might be enhanced by preventative chemotherapy against parasitic infection [21], and WASH improvements might disrupt parasite transmission [22–24]. So far there has been no effort to integrate these programmes, and little published evidence to support an understanding of the optimal composition, costs, and best practices behind such integration.

As early as 2012, the Ethiopian government wished to understand the situation with regards to parasite prevalence, WASH, and school feeding in schools and requested support from the Partnership for Child Development (PCD) and the United Nations World Food Programme (WFP) to design and implement a targeted, integrated pilot programme that would provide evidence for informed decision-making on SHN.

## Baseline national and regional surveys

**Fig 1:**
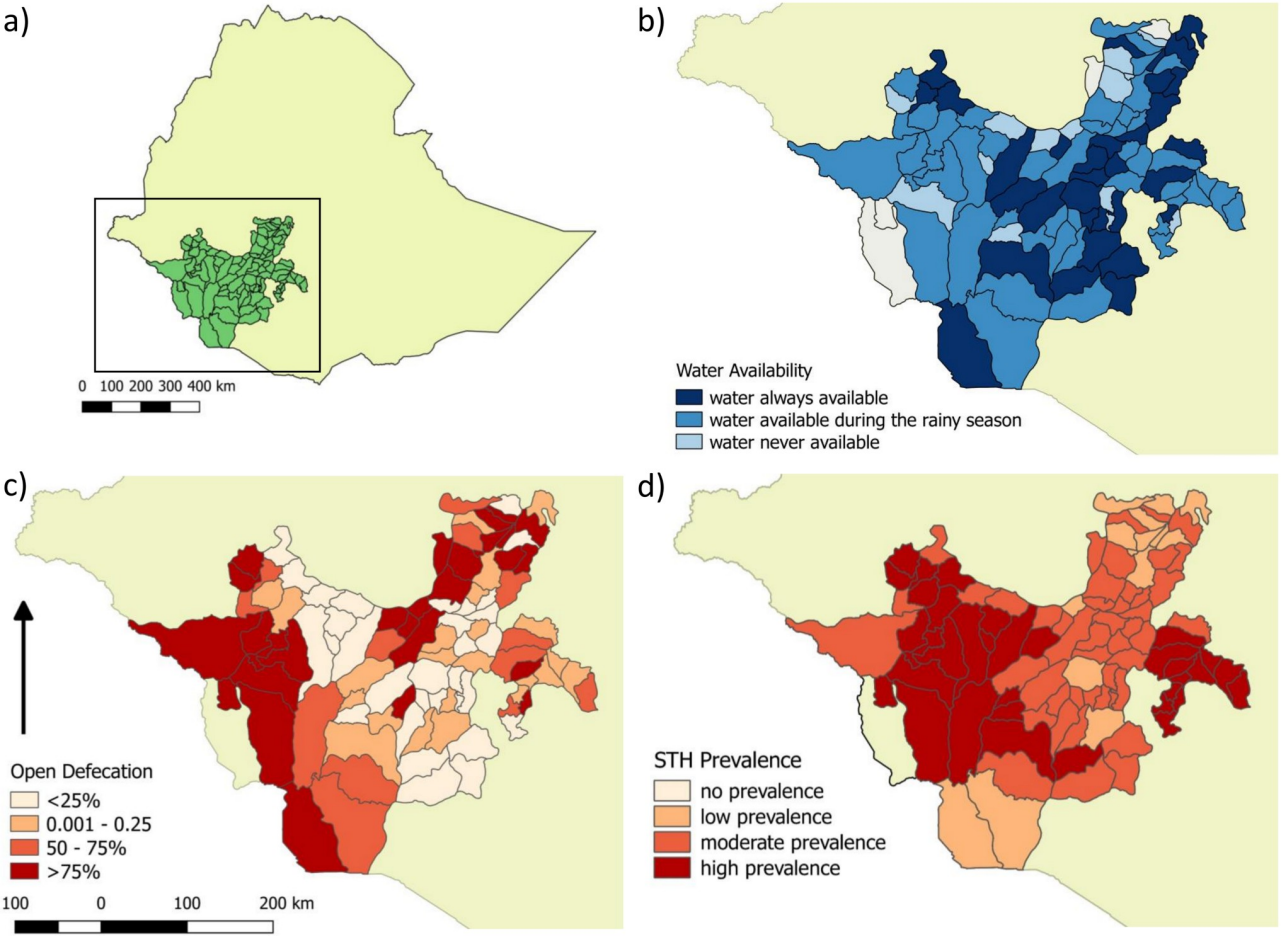
Maps depicting results from the regional survey in SNNPR. (a) Location of SNNPR in Ethiopia; SNNPR is indicated in the box. (b) Reported water availability. (c) Incidence of open defecation as a proportion. (d) STH prevalence according to WHO risk categories (no prevalence, low prevalence 1% to 20%, moderate prevalence 20% to 50%, high risk ≥50%) [32]. Maps created by the authors on Quantum GIS Geographic Information System. Open Source Geospatial Foundation Project, using baselayers accessed on www.gadm.org June 2016. SNNPR, Southern Nations Nationalities and People’s Region; STH, soil-transmitted helminths; WHO, World Health Organization.

An initial baseline analysis was conducted to assess the national-level prevalence and intensity of STH and schistosome infections, as well as to ascertain WASH indicators in 2,342 schools, involving 115,052 SAC across Ethiopia. The methodology behind, and results of, this mapping are published elsewhere [25]. The collected data have informed the national STH and schistosomiasis control programme, which was launched in 2015 with financial support from the Children’s Investment Fund Foundation (CIFF), the END Fund, the Department for International Development (DFID), and private funders and which leverages the global drug donation from pharmaceutical companies to combat these NTDs [19]. An analysis of schools surveyed in one particular region, Southern Nations, Nationalities and Peoples’ Region (SNNPR), showed inadequate WASH indicators against national guidelines [26], including inconsistent access to water in the region, no access to safe water sources in over 66% of schools and evidence of open defecation in 43% of schools Fig 1b and 1c. The same region also demonstrated a significant prevalence of intestinal parasites, estimated as 43% by the Kato-Katz method Fig 1d. These figures illustrate the poor socioeconomic and development indicators in SNNPR and the urgent need for control interventions that can support breaking the cycle of transmission.

## The integrated intervention

In response to the survey data, a multidisciplinary partnership was formed to pilot an evidence-based, integrated SHN programme in 30 schools in SNNPR, which targeted some of the key issues and could be combined with an ongoing school-feeding programme in the area. The programme was composed of three interventions: (1) daily provision of a hot meal to students via home grown school feeding (HGSF), an initiative that aims to deliver school feeding programmes using food that is procured from local farmers; (2) annual distribution of 400 mg of albendazole or 500 mg of mebendazole to all school children for STH (any individual testing positive for schistosome infection was administered with 40 mg/kg of praziquantel); (3) 15 of the schools received a WASH package, consisting of support for school WASH clubs to promote good hygiene practices amongst students as well as building or renovation of latrines and handwashing stations and provision of a water supply (see Grimes and colleagues, 2017, for a map of school locations [13]). Implementing partners were the Ethiopian government (Ministries of Education and Health) along with the WFP, the Schistosomiasis Control Initiative (SCI), and the Netherlands Development Organization (SNV). These interventions were designed to be complementary in their impact but also so that they could be implemented, monitored, and financed through a single, integrated mechanism.

## Operational research

The PCD, in partnership with the Ethiopian Public Health Institute (EPHI), as part of operational research, was involved in monitoring the integrated programme, which consisted of annual school feeding and WASH infrastructure surveys in each of the 30 schools as well as individual student assessments from a subset of 125 randomly selected students in each school. A total of 3,750 students provided stool and urine samples for assessment of STH and schistosome prevalence and infection intensities using the Kato-Katz method [27], or Hemastix Reagent Strips (Bayer HealthCare LLC, Indiana, USA). All selected students were measured for nutritional indicators, specifically individual heights, weights, and blood haemoglobin concentrations. A subsample of these students were randomly selected to answer a WASH knowledge, attitudes, and practices (KAP) questionnaire, and group interviews concerning knowledge and perceptions of SHN amongst community members, teachers, and school parents were coordinated in each school community. The research programme aimed to build the evidence base and identify best practices in integrating interventions to inform programme decision makers at the regional, national, and global levels. The programme also offered a unique insight into specific synergies as a result of integration and into the efficiencies in integrating implementation.

## Initial findings

The research surrounding the work combines both qualitative and quantitative analyses to develop a complete understanding of the needs, costs, and impact associated with such a design, as well as how to ensure effective implementation. The in-depth analysis of baseline data from the ESHI schools is presented in Grimes and colleagues [13], which found STH prevalence to be 23% among the children sampled according to single-Kato-Katz assessment; almost one quarter of children were suffering from nutritional deficiencies, including anaemia (23%), stunting (28%), and wasting (14%), demonstrating the necessity for a targeted health intervention. Multiple efficiencies of integrating these interventions into program design were shown, with significant cost-saving opportunities identified around programme planning and monitoring and evaluation activities [28]. From the analysis on costs, it was also evident that communities made a significant contribution to the programme, primarily in the form of provision of firewood, building materials, and labour for the construction of school kitchen and canteens. Although these contributions indicate the value that communities place on schools and on provision of schools that can support the health of children, it also highlights the need for implementers to be wary of overburdening already impoverished communities when considering programme design. This was a concept explored further through analysis of group interviews [29].

## Mainstreaming and scale-up of SHN

Schools provide an existing and pervasive infrastructure from which to launch health programmes, reducing start-up costs, accelerating programme implementation, and reducing programmatic costs, whilst at the same time optimizing the benefits for education, increasing access to care for the most marginalized, and encouraging girls to attend and stay in school [30]. As an achievement of the MDGs, more children are now attending school than ever before [8, 9]. With the shift to the Sustainable Development Goal (SDG) era, the education sector is well placed to take a role in the health of the school-going population, ensuring key health interventions and messages reach the remote, rural, ‘end of the road’ populations who are currently missed by overstretched health systems while at the same time supporting achievement of equitable education.

However, governments require evidence to cost, design, and implement SHN programmes that meet the multiple and diverse health and education needs of their target communities. ESHI provides empirical evidence, the first of its kind, of the costs and efficiencies of integrating multiple health interventions onto a school platform, as well as providing a framework from which to integrate multiple school health interventions in a resource-efficient manner. Advocacy and evidence surrounding the operational research has translated into strengthened institutional capacity and improved cross-sectoral coordination for SHN in Ethiopia. The identification of synergies and best practices as part of Ethiopia’s overall SHN strategy will support the government of Ethiopia in moving forward with its SHN agenda.

Preliminary results after four years of implementation indicate improvements in key indicators for child health and nutrition, including STH prevalence and intensity, hand washing, and open defecation. Ethiopia has a net enrolment rate of over 100% in primary education, with ambitious targets to reduce drop out and similarly increase secondary education enrolment from 64% in its next strategy of educational development [16]. In a win–win situation for health and education, these significant and growing proportions of Ethiopian SAC enrolling in school provides an easily accessible population to target health and nutrition interventions, as well as in the act of providing incentives for children to remain in school and learn whilst there.

Although final results from the programme are forthcoming, they indicate decreases in prevalence and intensity of STH and improvements in hygiene and sanitation behaviour amongst the school children. The experience of ESHI has led to government backing for scale-up of SHN in Ethiopia, many of the fundamentals of ESHI are now being used as a model for regional and national plans, and both Ministries of Health and Education are now pledging continuing support for SHN programmes, set to benefit 25 million SAC across the country [31].

The sustainability of national school health programmes depends on mainstreaming SHN programmes into national policies and plans, as well as increasing national financing for SHN and strengthening cross-sectoral institutional implementation capacity. These programmes, when well designed and based on evidence, serve to promote both health and education, thereby supporting progress towards the SDGs.

The strategy adopted by the Ethiopian government is leading the way in multidisciplinary, multisectoral programming, providing a model that can be adapted and adopted by other countries. Cost efficiencies and synergistic benefits of integration provide a mechanism to address pressing health issues of SAC. ESHI provides real-world evidence of the related costs and synergies of integration for programmes seeking to address health and educational outcomes for SAC in Ethiopia and serves as a reference point for other countries looking to scale up targeted SHN interventions.
